# Towards High-Accuracy Athletic Injury Predictions Using a First-Principles Modelling Approach: Theory to (Future) Practice

**DOI:** 10.1007/s40279-026-02435-2

**Published:** 2026-05-09

**Authors:** Judd T. Kalkhoven, Franco M. Impellizzeri, Dean L. Norris, W. Brent Edwards

**Affiliations:** 1 School of Health Sciences, Western Sydney University, Campbelltown Campus, Narellan Rd & Gilchrist Dr, Campbelltown, NSW 2560 Australia; 2 Human Performance Research Centre, Faculty of Health, University of Technology Sydney, Sydney, NSW Australia; 3 Human Performance Laboratory, Faculty of Kinesiology, University of Calgary, Calgary, AB Canada

## Abstract

Athletic injury has previously been defined as tissue damage or other derangement of normal physical function due to participation in sports, resulting from rapid or repetitive transfer of kinetic energy. Alternatively, athletic injury has also been described as occurring when the stresses and strains experienced by a tissue result in damage severe enough to be considered an athletic injury. In mechanical models quantifying damage and the accumulation of damage over time, damage is commonly represented using a damage variable (*D*) ranging between 0 and 1, where *D* = 0 corresponds to an undamaged state and *D* = 1 corresponds to complete mechanical failure. Adopting this approach, a mathematically deterministic definition for athletic injury can be established, allowing precise predictions of athletic injury occurrence in mathematical models. Specifically, an athletic injury can be mathematically defined as occurring when the damage (*D*) experienced by a tissue exceeds a critical damage threshold (*D*_c_), that is, *D* > *D*_c_, with complete tissue failure occurring at a damage variable of 1. Alternatively, athletic injury can also be mathematically defined as an applied mechanical load (*L*) exceeding a critical tissue strength threshold (*S*_c_), that is, *L* > *S*_c_, with complete tissue failure occurring when *L* > failure strength (*S*_f_). While determinism is valuable for establishing a precise definition of athletic injury for mathematical models, in practice, probabilistic models are needed to account for the inherent variability and uncertainty in estimating the primary variables determining athletic injury outcomes. By examining the overlap of probability density functions of tissue load and strength estimations, or the probability that *D* > *D*_c_, the probability of an athletic injury occurring can be appropriately quantified. However, to offer practical utility for the prevention of athletic injuries in real-world settings, athletic injury predictions must provide sufficient windows for intervention. For injuries involving sudden unanticipated loads that exceed the possible physiological ranges of tissue strength, this method of athletic injury risk assessment may be of little value, as there is no window of opportunity for intervention. In scenarios where future loads experienced by an athlete can be estimated, tissue damage accumulates over time, or tissue load and strength values converge over time, actualising this approach by obtaining accurate estimations of these variables is likely crucial for achieving high-accuracy athletic injury predictions that offer specific data-driven windows for intervention.

## Key Points

**Table:** 

The development of accurate, actionable, and transportable athletic injury prediction models requires a conceptually robust and logically coherent theoretical definition of athletic injury—one that can be formalised, operationalised through objective criteria, and mathematised for integration into predictive models.	
At the clinical or diagnostic level, injury may be best represented as a bodily lesion, as reflected in definitions from the World Health Organization (WHO) and Centers for Disease Control and Prevention (CDC). However, for the purposes of predictive modelling, simulation, and causal inference, injury is more appropriately operationalised as a logico-mathematical construct that can be effectively integrated into probabilistic models of tissue damage accumulation, lesion formation and progression, tissue failures, and injury risk.	
While not a formal requirement of prediction, aligning data collection more closely to the most fundamental and mechanistically relevant causal structures determining athletic injury outcomes—specifically tissue load, strength, and damage interactions—may reduce noise and improve the performance and transportability of prediction models. However, to accurately quantify injury risk, appropriate probabilistic models that account for the inherent variability and uncertainty in estimating these critical variables are required.	
By obtaining more direct assessments of tissue strength and damage, potentially by harnessing advancements in scanning technologies, artificial intelligence, and mechanical testing, the effects of all-cause tissue degradation may be accounted for. This may assist with bypassing modelling through the mechanical loading and damage accumulation (and removal, i.e., tissue remodelling and repair) pathway, avoiding the rapid error propagation associated with this modelling approach. For certain injury types, the successful implementation of this approach may provide powerful athletic injury risk assessments that offer specific data-driven windows for intervention.	

“In any special doctrine of nature, there can be only as much proper science as there is mathematics therein”—Immanuel Kant [1]

## Introduction

In the realm of competitive sports, the prevention of athletic injuries is considered paramount for the longevity and well-being of athletes and the overall success of sports teams and individual performances. However, despite its widely recognised importance and an extensive body of research on the topic, the regular occurrence of sports injuries is persistent. In some contexts, injury rates have remained relatively stable or increased over several years, as seen with hamstring injuries [2, 3]; encouragingly, some improvements in injury rates have been observed in others [4]. Nevertheless, injuries continue to be a major concern for athletes, practitioners, and sporting organisations. To assist, it is widely considered that prognostic models predicting athletic injury occurrences may offer considerable practical utility for their prevention, assuming an appropriate opportunity for intervention exists. While this is not a certainty, and impact studies examining the effect of any prognostic model on athletic injury outcomes would ultimately be needed, owing to its great potential, prediction has been labelled “The Modern-Day Sport-Science and Sports-Medicine ‘Quest for the Holy Grail’” [5]. However, the dynamic and complex nature of sports activities, coupled with suboptimal methodological approaches [6, 7], has proven to be a major obstacle in the ‘quest’ for accurate, actionable, and transportable (Table 1) athletic injury predictions.

**Table 1:** Nomenclature and key definitions used in this article

Terms	Operational definitions
Accurate and actionable	In prognostic models, accuracy refers to the proportion of correct predictions made by a prediction model, encompassing both true positives and true negatives, relative to the total number of predictions. Accordingly, it assesses how well the model correctly identifies outcomes across all possible instances. However, in the context of athletic injuries, and clinical contexts in general, accuracy as a performance metric does not fully capture the model's performance. To avoid any confusion, and with full recognition of the various performance metrics that exist for prognostic models, when predictions are described as ‘accurate and actionable’ in this article, this refers to prognostic models that exhibit strong predictive performance in terms of both discrimination and calibration, while also providing meaningful opportunities for intervention
Anisotropic	Anisotropic refers to a material property or characteristic that varies depending on the direction of measurement. In other words, an anisotropic tissue exhibits different mechanical, thermal, or electrical properties along different axes or directions due to its internal architecture or composition
Athletic injury	Tissue damage and loss of physical function during sports participation, resulting from singular, sustained, or repetitive transfer of mechanical energy, where the damage experienced is not a normal part of the physical training and positive adaptation process, but exceeds the threshold of mechanical and physiological tolerance. This is dependent upon the nature and degree of tissue damage sustained
Causal directed acyclic graph (DAG)	A causal DAG is a graphical tool used to represent causal relationships. In a causal DAG, nodes represent variables (such as factors or outcomes), and directed edges (arrows) represent causal influences from one variable to another. The ‘acyclic’ aspect means that the graph does not contain any cycles, implying that causality is not circular
Causal graph	A causal graph is a visual representation of the causal relationships between variables, typically depicted as a directed acyclic graph (DAG)
Causal inference	Causal inference refers to the science of determining cause-and-effect relationships. It involves using statistical methods and research designs to draw conclusions about causality by controlling for various forms of bias [22, 27, 31]
Causal Markov condition	In causal inference, the causal Markov condition (often referred to as the causal Markov assumption) is foundational. It states that, in a correctly specified causal graph, each variable is conditionally independent of its non-descendants, given its direct parents (i.e., its direct causes) [22, 27, 31]. In other words, once the values of a variable’s direct causes (its direct parent nodes) are known, additional information about other variables that are not downstream (i.e., not its effects) offers no further predictive power. This is because all relevant statistical information required for predicting the variable is already contained in its direct parent variables [22, 27, 31]
Deterministic models	These models predict outcomes with complete certainty, assuming all variables and their interactions are known and measured precisely. They do not account for variability or uncertainty, e.g., *Y* = *f*(*X*), and therefore serve primarily as theoretical ideals, since such complete information is unattainable in real-world settings
Direct cause	In causal inference, a direct cause refers to a variable that directly influences another variable without any intermediate variables mediating the relationship. It represents the direct causal pathway from the cause to the effect
Falsifiability	Falsifiability is a fundamental criterion in the scientific method, referring to the degree to which a hypothesis or theory can be shown to be false through observation or experimentation. A falsifiable theory must make precise, testable predictions that may be contradicted by empirical evidence. As philosopher Karl Popper [9] argued, if a theory cannot be tested or potentially refuted in this way, it does not qualify as scientifically valid and instead begins to fall into the realm of pseudoscience. This is because a theory that cannot be invalidated is immune to critical evaluation It is important to note that this principle is not without controversy. Falsifiability is often seen as an ideal rather than an absolute requirement, as real-world scientific testing rarely yields definitive refutation. Instead, theories are generally subjected to repeated testing, and scientists aim for theories with a high degree of falsifiability, continually refining them as new evidence emerges
Formalisation	Formalisation is the process of expressing ideas, concepts, or systems in a precise, structured, and standardised form, often using symbols, rules, and formal logic to ensure clarity and consistency
Indirect cause	An indirect cause, also known as a mediated cause, is a variable that influences another variable through one or more intermediate variables in a causal pathway. It represents the causal effect that is transmitted through one or more mediators
Mediation	Mediation refers to the process through which an independent variable influences an outcome variable indirectly via one or more intervening variables, known as mediators. These mediators explain the mechanism or pathway by which the initial variable exerts its effects on the outcome, providing insights into the underlying causal chain. This is distinct from moderation, which concerns factors that alter the strength or direction of the relationship rather than transmitting the effect
Mechanical hysteresis	Mechanical hysteresis relates to a lag between the applied force and resulting deformation in a tissue, characterised by a loop in the stress–strain curve during loading and unloading cycles. This indicates energy loss within the tissue due to internal friction or other dissipative processes
Mechanical loading	Mechanical loading refers to the external force or combination of forces applied to a tissue, causing stresses and strains. Depending on the nature and direction of the applied forces, loading can come in a variety of modes (e.g., tension, compression, shear, bending, or torsion)
Mechanical stiffness	Mechanical stiffness is a measure of a tissue’s resistance to deformation under an applied force. It quantifies how much a tissue deforms when subjected to a given load, with higher stiffness indicating less deformation
Mechanical strain	Strain is a normalised measure of tissue deformation expressed as the ratio of deformation to the initial dimensions. Two types of strain exist: normal strain, which is related to changes in size, and shear strain, which is related to changes in shape. Normal strain may be tensile or compressive depending on the type of loading
Mechanical strength	In the context of athletic injury, mechanical strength refers to the ability of a tissue to withstand and resist applied forces without undergoing significant breakage or failure
Mechanical stress	Stress is defined as the internal force per unit area that develops within a tissue in response to an applied force. Stress may be characterised as normal (force perpendicular to a plane) or shear (force parallel to a plane). Normal stress may be tensile or compressive depending on the mode of loading
Mechanical testing	Mechanical testing refers to the evaluation of a tissue’s mechanical properties, such as strength, elasticity, hardness, and toughness, by applying controlled forces or loads
Palmgren–Miner rule (or Miner’s rule)	The Palmgren–Miner rule (or Miner's rule) is a linear cumulative damage rule used to predict the fatigue life of materials and tissues under varying stress conditions [48, 49]. It states that the total damage (D) in a material or tissue subjected to cyclic loading is equal to the sum of the ratios of the number of applied cycles (n_i_) to the number of cycles to failure (N_i_) at each stress level: $D= \sum_{i=1}^{k}\frac{ni }{Ni }$
Predictability	Predictability refers to a theory’s ability to generate specific, testable predictions about future observations or experiments. It implies that the theory should outline what outcomes are expected under certain conditions and what results would contradict the theory. Predictability is crucial for falsifiability, as it establishes clear criteria for testing and determining whether the theory can be refuted, thereby making it scientifically meaningful. Without predictability, a theory cannot be tested and, thus, cannot be falsified [9]
Probabilistic models	These models predict outcomes by considering the inherent variability and uncertainty in data and measurements. They provide a range of possible outcomes and their associated probabilities, rather than a single, definite prediction, e.g., *P*($Y\left| X \right.$) indicating the probability of *Y* given *X*
Reproducibility	Reproducibility is the extent to which consistent results can be obtained using the same methods, data, and conditions when an experiment or study is repeated by different researchers or at different times
Tissue damage	Tissue damage refers to the disruption of the molecular bonding that maintains tissue integrity and functional capacity, resulting from the transfer of energy, such as mechanical, radiant, thermal, chemical, or electrical energy. However, while this bond-level damage plays a central causal role in tissue degeneration and failure, in the absence of overt manifestations of structural disruption (e.g., cracking or tearing), tissue damage is commonly unobservable. Instead, tissue damage must be inferred from indirect indicators such as measurable changes in tissue-level mechanical properties or imaging markers Notably, the physically observable manifestations of tissue damage vary by tissue type. For example, microcracks, diffuse damage, and cracking in bone [50]; collagen unfolding, kinked fibres, and tearing in tendons [38, 51]; and sarcomere disruption and fibre tears in muscles [52, 53]
Transportability	Refers to the extent to which findings from a study conducted in one population can be applied to a different external target population [22, 27, 31]
Weibull distribution	The Weibull distribution is a continuous probability distribution widely used in materials science, reliability engineering, and failure analysis to model the variability in material strength, fatigue life, and the likelihood of failure under stress [54]. It is particularly effective for describing the probability of failure for brittle materials, where failure is locally initiated by the presence of defects or flaws

In the pursuit of predicting athletic injuries, many challenges exist. At the forefront is the lack of a conceptually robust and logically coherent theoretical definition of athletic injury [8]. This is important because a theoretical definition provides the fundamental framework through which a concept can be understood scientifically and appropriately operationalised [9–17]. Without a sufficiently coherent definition, the theoretical and methodological formalisation (Table 1) of athletic injury and associated constructs, as well as the development of more accurate operationalisations and appropriate mathematical models, is hindered [8–11, 13, 15, 17–21]. Moreover, mathematising such a definition allows athletic injury to be represented more precisely in predictive models, simulations, causal inference systems, and formal validation frameworks [8, 11, 15, 16, 22, 23]. Collectively, advancements in these areas improve the clarity, falsifiability, reproducibility, and scientific utility of athletic injury research (Table 1) [8, 9, 24–26].

A second potential reason for the lack of progress in athletic injury prediction is that currently collected data may be too far removed from the phenomenon of athletic injury to contain sufficient mechanistic signal for reliable predictions [22, 27, 28]. Although not a strict requirement of prediction, aligning data collection more closely to the fundamental causal structures, processes, and critical variables determining athletic injury outcomes may improve the quality and performance of prediction models [22, 27]. This approach offers several benefits: relevant causal variables can be identified and prioritised as predictors, irrelevant or redundant variables can be excluded, and critically, the transportability of models across different populations, settings, or sporting contexts may be enhanced [22, 27, 29, 30]. Adopting a causal framework may also provide important insights into how modifying a (causal) predictor (i.e., intervention) will impact the outcome of interest [22, 27, 31, 32].

For athletic injuries, current understanding emphasises that the most fundamental (ultimate) causal variables centre around the mechanical loads that are applied to a tissue, a tissue’s capacity to withstand these loads (i.e., mechanical strength, Table 1), and the accumulation of tissue damage (Table 1) over time [8, 32–39]. Accordingly, quantifications of these variables, along with the establishment of a mathematically precise definition of athletic injury, can enable the development of robust mathematical models for athletic injury prediction. However, while determinism is valuable for clearly defining a concept for mathematical modelling, purely deterministic models (Table 1) may, depending on the context, represent an impractical ideal in real-world settings, as they commonly do not consider the inherent variability and uncertainty [40, 41]. In such cases, probabilistic models (Table 1) are necessary [40, 41]. For example, uncertainty in quantifying tissue-specific loading, mechanical strength, and the accumulation of tissue damage over time necessitate probabilistic approaches when using these variables for athletic injury predictions.

In light of the ongoing prevalence of sports injuries, and the potential for high-performing athletic injury prediction models to impact the sporting landscape, this article has four main aims: (1) to briefly outline the need for accurate and actionable athletic injury predictions; (2) to provide a conceptually and logically coherent fundamental theoretical definition of athletic injury, and to mathematise this definition for utilisation in predictive modelling; (3) to apply this definition and understandings of causality to simulate real-world scenarios of athletic injury occurrence, demonstrating how probabilistic models based on critical causal variables can reduce complexity and assist with achieving accurate, actionable, and transportable athletic injury predictions; and (4) to bridge the gap between theory and practice by exploring potential avenues for the real-world actualisation of the explored approach for effective probabilistic predictions of athletic injury occurrence in applied sporting contexts.

This involves examining the potential implications and limitations of modelling through the mechanical loading and damage accumulation (and removal, i.e., tissue remodelling and repair) pathway, as well as exploring alternative approaches that bypass modelling through this pathway by leveraging scanning technologies, artificial intelligence, and mechanical testing to obtain more direct estimates of tissue strength and damage. By addressing each of these aims, and working from first principles forward, this article is intended to provide an explicit framework for achieving high-accuracy athletic injury predictions that enable specific, data-driven windows for intervention for certain types of injury [32]. Additionally, this article highlights that, for certain injury types, achieving highly accurate and reliable predictions likely depends on how adequately the collected data capture or reflect a select few critical variables determining athletic injury outcomes. In other words, through an educated understanding of the system at hand, the prediction of certain injuries can be reduced to a problem in measurement and estimation of these key variables, rather than advanced data analytics or complex systems, despite the latter being a common emphasis in the sports science and medicine literature [42–47].

## Nature of the Problem: The Need for Accurate and Actionable Athletic Injury Prediction Models

For a prediction model to effectively support athletic injury prevention, it must not only estimate injury risk with the necessary accuracy for informed decision making, but must also offer sufficient opportunities for intervention. This does not ensure injury prevention; prevention is mostly dependent upon the effectiveness of the interventions applied in response to any predictions. Regardless, producing accurate athletic injury predictions that enable appropriate opportunities for intervention is, in itself, a challenging endeavour. Within the sporting domain, the development of an athletic injury prediction model that is high-performing, appropriately validated, and transportable between sporting contexts, has yet to occur [6]. This is problematic since, aside from the more obvious and well documented negative effects of athletic injury on physical and psychological health [55–57], career trajectories [58, 59], and athlete and organisational finances [60–62], many current approaches to athletic injury prevention are not without a cost to athletes and sporting organisations, that is, a trade-off exists. For example, training load management strategies aiming to reduce injury risk often involve the restriction or cessation of training and competitive match exposures, which can be a source of conflict among relevant stakeholders and can have negative consequences on sporting performance, which is the primary outcome of importance for elite athletes. If predictions are weak and therefore incapable of effectively assessing injury risks, interventions based on these predictions may be harmful to athletes, for example restricting an individual’s participation when it would be more beneficial for them to train or compete. Indeed, when such interventions are imposed on athletes against their will, and without robust predictions as a justification, ethical concerns regarding the appropriateness of these approaches become increasingly evident. This underscores the pressing need for high-performing athletic injury prediction models; if predictive capabilities are low, athletes may be unnecessarily withheld from valuable training or competitive opportunities, impeding their progress and potentially affecting their career trajectories. If predictive capabilities are high, the model may serve as a valuable tool for making informed decisions for injury prevention, minimising any potential negative impacts on an athlete’s training and competitive goals.

## Defining Athletic Injury: Theory to Mathematisation

### Theoretical Definition

Definitions can be applied at both the theoretical and operational levels, with each having distinct purposes and applications. A theoretical (fundamental) definition is important as it establishes a core, conceptual understanding of a term, concept, or phenomenon [9–16]. Through this, theoretical definitions provide the essential conceptual framework from which appropriate operational definitions may be developed, and the limitations of any chosen operational criteria properly understood [9, 11–16, 18, 63]. Various attempts to theoretically define an athletic injury have been made [64–67], with the International Olympic Committee (IOC) currently defining sports (athletic) injury as:

*“Tissue damage or other derangement of normal physical function due to participation in sports, resulting from rapid or repetitive transfer of kinetic energy”* [67].

#### Definition 1

Alternatively, athletic injury has also been described as occurring when the stresses and strains (Table 1) experienced by a tissue result in damage severe enough to be considered an injury [33, 34]. Note that these descriptions are partially reflective of one another, with the area under a stress–strain curve representing the energy absorbed during deformation, which is sometimes (but not exclusively) due to a transfer of kinetic energy. Regardless, an important distinction worth highlighting here surrounds understandings of tissue damage. Some degree of tissue damage is an unavoidable part of the physical training process [68–70], and may act as an important stimulus for tissue remodelling and adaptation [71–74]. In addition, it can also be argued that various forms of fatigue (e.g., neuromuscular) constitutes a form of “other derangement of normal physical function.” Reasonably, these conditions should not be considered an athletic injury, as every athlete would then inevitably incur an injury shortly after commencing their training, an outcome that is clearly unreasonable. Rather, an athletic injury more accurately occurs when the tissue damage experienced exceeds some critical damage threshold, whereby the damage sustained is not a normal part of the physical training and positive adaptation process [8, 32, 35]. To better reflect these distinctions and build upon the definition provided by the IOC, a new theoretical definition for athletic injury is proposed:

“*Tissue damage and loss of physical function during sports participation, resulting from singular, sustained, or repetitive transfer of mechanical energy, where the damage experienced is not a normal part of the physical training and positive adaptation process, but exceeds the threshold of mechanical and physiological tolerance. This is dependent upon the nature and degree of tissue damage sustained*.”

#### Definition 2

This updated definition provides a more accurate description of athletic injury, aligning more closely with the broader concept of injury as defined by various global authorities such as the World Health Organization (WHO) [75], Centers for Disease Control and Prevention (CDC) [76], the International Classification of External Causes of Injury (ICECI) [76], and the International Classification of Diseases (ICD-11) [77], all of which emphasise a demarcating threshold of bodily harm to distinguish injury from non-injury (Table 2).

**Table 2:** (General) injury definitions from global authorities

World Health Organization (WHO) [75]	A bodily lesion at the organic level, resulting from acute exposure to energy (mechanical, thermal, electrical, chemical, or radiant) in amounts that exceed the threshold of physiological tolerance
Centers for Disease Control and Prevention (CDC) and the International Classification of External Causes of Injuries (ICECI) [76]	A (suspected) bodily lesion resulting from acute overexposure to energy (mechanical, thermal, electrical, chemical, or radiant) interacting with the body in amounts or at rates that exceed the threshold of physiological tolerance
International Classification of Diseases (ICD-11) [77]	Physical or physiological bodily harm resulting from the interaction of the body with energy (mechanical, thermal, electrical, chemical, or radiant, or due to extreme pressure) in an amount, or at a rate of transfer, that exceeds physical or physiological tolerance. Injury can also result from a lack of vital elements, such as oxygen. Poisoning by, and toxic effects of, substances are included, as is damage to or due to implanted devices

The following sections are dedicated to clarifying potential conceptual ambiguities within the newly proposed definition of athletic injury (Definition 2) and to exploring its appropriate operationalisation. However, a comprehensive justification for this definition, grounded in the principles of Aristotelian essentialism, classical logic, and Carnapian explication is provided in full elsewhere [8]. This work also provides a detailed rationale for why symptoms such as pain and swelling, as well as availability for sports participation, are unsuitable inclusions for a theoretical definition of athletic injury.

### Nature and Degree of Tissue Damage Sustained

Within the proposed definition of athletic injury (Definition 2) “nature and degree of tissue damage sustained” refers to the specific characteristics, properties, or type of tissue damage that distinguishes an athletic injury from normal responses to physical training. It encompasses both the qualitative aspect (i.e., the type of microstructures within specific tissue types affected) and the quantitative aspect (i.e., the extent or severity of the damage sustained).

An illustrative example highlighting the importance of considering the nature of tissue damage sustained is the distinction between muscle damage and muscle injury, which are distinct clinical entities [52]. In some contexts, muscle damage is a largely unavoidable and normal part of the physical training process [68–70] that commonly precedes positive adaptations such as the repeated bout effect [78], and increased hypertrophy and strength (although the causal nature of this relationship has been questioned [74, 79]). It is characterised by sarcomere dissolution and damage to various intramuscular microstructures, for example, desmin disruption and catabolism, Z-disk streaming, and other cytoskeletal or membrane alterations, etc. [52, 53]. Given its frequent occurrence during and after training, and the beneficial adaptations that commonly ensue, reasonably, muscle damage should not be classified as an athletic injury. Rather, muscle injury more accurately occurs when there are structural tears in muscle fibres [52], which provides no adaptive benefit and typically requires long and incomplete recovery processes [52].

The significance of considering the degree of tissue damage is exemplified by the distinction between mechanical fatigue of bone and the development of bone fractures. Mechanical fatigue, characterised by a temporary reduction in bone stiffness and strength, is a stimulus for positive bone adaptation in accordance with Wolff’s Law [71–73, 80, 81]. In this context, the microstructural changes that occur are part of a normal physiological process that strengthens bone over time [71–73, 80, 81]. Conversely, the formation of bone cracks or fractures represents a pathological outcome [82], that is, the exceedance of mechanical and physiological tolerance, and therefore an injury in this context.

### Mathematisation

In mechanical models quantifying the accumulation of damage over time, damage is commonly represented using a damage variable (*D*) ranging between 0 and 1, where *D* = 0 corresponds to an undamaged state and *D* = 1 corresponds to complete mechanical failure (i.e., an inability to carry load) [35, 48, 83]. Adopting a similar approach, the proposed theoretical definition for athletic injury can be mathematised, allowing for precise predictions of athletic injury occurrence in mathematical models. Specifically, an athletic injury can be mathematically defined as:

$$D > D_{{\mathrm{c}}}$$

#### Definition 3

In Definition 3, first proposed by Edwards [35], an athletic injury occurs when the damage (*D*—quantified between 0 and 1) sustained by a tissue is greater than a critical damage threshold (*D*_c_—also quantified between 0 and 1), that is, *D* > *D*_c_. While mechanical models typically determine damage accumulation based on the mechanical loading pattern experienced by a structure [35], in the context of athletic injury, damage includes both tissue damage due to loading and any alterations in tissue damage induced by physiological processes, such as remodelling and repair [84]. This is particularly relevant to athletic injuries exhibiting a gradual onset mechanism, whereby significant damage removal can occur during periods of rest and recovery [85, 86].

Under the assumption that the critical damage threshold (*D*_c_) is exceeded by damage inflicted from a single applied load, either from an acute loading event (acute injury) or a final applied load at the end of a series of damaging loads (gradual onset injury), an athletic injury can also be mathematically defined as follows:

$$L > S_{{\mathrm{c}}}$$

#### Definition 4

In Definition 4, an athletic injury occurs when an applied mechanical load (*L*) exceeds a critical tissue strength threshold (*S*_c_), with complete tissue failure occurring when* L* is greater than the failure (ultimate) strength (*S*_f_) of a tissue (i.e.,* L* >  *S*_f_). Note that applied loads result in stresses and strains that damage a tissue, while the mechanical strength of a tissue determines its resistance to load, damage, and failure. Accordingly, this definition simply provides an alternative conceptualisation of the same physical phenomenon and is mathematically equivalent to Definition 3. In fact, these two definitions are mathematically related through the concept of residual strength (*S*_r_), which describes a tissue’s loading capacity as a function of damage accumulation:

1 $$S_{{\mathrm{r}}} = S_{{\mathrm{f}}} \times \left( {1 - D} \right)$$

#### Loss of Physical Function

While Definitions 3 and 4 are mathematically related through the concept of residual strength, loss of physical function, as conceptualised within the proposed theoretical definition of athletic injury (Definition 2), is also intended to operate within this same mathematical framework. Here, a loss of physical function specifically refers to a deterioration in the (objectively measurable) mechanical functioning of the tissue (e.g., load bearing capacity, stiffness, elasticity etc.), which are functions of the original state of a tissue, and any tissue damage present. Accordingly, tissue strength, damage, and physical function all provide alternative conceptualisations of the same physical phenomenon and exist within a unified physics-based framework. While the relationship between localised damage and mechanical properties may become decoupled at the structural level, this is a function of scale, due to scale-dependent emergent behaviours such as stress redistribution and deformation. To address this complexity and increase precision, practical engineering approaches like finite element modelling and continuum damage mechanics (Table 1) are commonly utilised to model the effects of localised damage on mechanical behaviours [87, 88].

### Setting Critical Tissue Damage or Strength Thresholds

Identifying relevant critical damage thresholds that are reflective of the proposed theoretical definition for athletic injury is a difficult task. In materials sciences, such thresholds are typically determined through material testing protocols, which commonly involve the application of various forms of stress (either singular or repetitive) to assess the mechanical behaviour of a material under load. Through this process, relevant thresholds can be determined based on empirical evidence supporting the applicability of a particular threshold relative to the intended use or function of a structure or material. Then, in practice, this threshold can be estimated using predictive models.

In applied sporting scenarios, selecting an exact critical damage threshold may, in some contexts, be a challenging endeavour, with such a threshold likely relying upon the triangulation of empirical evidence to supports its relevance. For instance, this may involve an analysis surrounding the degradation of physical function, that is, mechanical properties such as stiffness, elasticity, and strength, that commonly accompany damage accumulation, the physical manifestation of damage (which varies between tissues, e.g., cracking in bone [50], kinked fibres and tearing in tendon [51], etc.), and the future recovery and remodelling of the tissue, that is, the adaptations that ensue. In certain scenarios, the setting of a relevant threshold may be relatively straightforward. For example, the physical manifestation of tissue damage, such as the development of cracks (or a certain degree of cracking) in bone, may serve as an appropriate tissue injury threshold, with this physical manifestation corresponding with a particular mathematical tissue damage threshold between 0 and 1. If a researcher is interested in complete tissue failure, such as tendon rupture or bone fracture, the damage threshold can be set to 1, which also represents the exceeding of the failure strength (*S*_f_) of a tissue and a complete loss of functional capacity (i.e., an inability to tolerate load). Ultimately, however, due to the considerable differences that exist between tissue types, precise operationalisations tailored to specific tissue types (i.e., defining critical damage thresholds for bone, tendon, muscle etc.) are needed.

## Leveraging Causality in the Quest for Athletic Injury Prediction: Causal Inference and the Causal Markov Condition

Considering the multifactorial and complex nature of sports injury occurrence, there have been widespread calls for the adoption of complex systems and system dynamics approaches to injury prediction [42–47, 89], alongside complementary frameworks such as the ‘Web of Determinants’ [47]. While such perspectives may hold merit in certain contexts, overly complex approaches can also prove problematic, often requiring the integration of an overwhelming number of poorly understood variables and interactions. This can inflate noise-to-signal ratios, hinder interpretability, and lead to unnecessarily complicated, unstructured, and resource-intensive models. Furthermore, while dynamic and complex systems are often referenced as essential frameworks in the sports medicine literature, there appears to be a lack of formal application or detailed modelling (i.e., formalisation and parametrisation) in the context of athletic injury prediction and prevention.

To assist, and while not strictly a requirement of prediction, understandings of causality can provide a particularly strong framework for prediction in certain contexts, reflecting the underlying mechanisms driving the relationships between variables [22, 27, 31]. By aligning data collection more closely to the causal structures, processes, and critical variables determining athletic injury outcomes, complexity can be reduced, unnecessary data inclusions avoided, and the performance of predictive models improved [22, 27, 31]. This approach offers various benefits: relevant causal variables can be identified and selected as predictors, model noise can be minimised, and the transportability of models can be enhanced [22, 27, 29–31]. Adopting a causal framework may also provide important insights into how modifying a (causal) predictor (i.e., intervention) could impact the outcome of interest [22, 27, 31, 32]. Importantly, various perspectives to causality exist (e.g., structural causal models, potential outcomes etc.) with this article adopting a structural causal model perspective relying on causal directed acyclic graphs (DAGs; Table 1) [22, 27, 31, 32]. However, the conceptual approach presented in this article can be applied to other frameworks as well [90].

In causal inference, the causal Markov condition (often referred to as the causal Markov assumption; Table 1) is foundational. It states that, in a correctly specified causal graph (Table 1), each variable is conditionally independent of its non-descendants, given its direct parents (i.e., its direct causes) [22, 27, 31]. In other words, once the values of a variable’s direct causes (its parent nodes) are known, additional information about other variables that are not downstream (i.e., not its effects) offers no further predictive power. This is because all relevant statistical information required for predicting the variable is already contained in its direct parent variables. The causal Markov condition is particularly useful for simplifying both causal and predictive models by focusing on the most immediate causes of an outcome, rather than accounting for a comprehensive history of past or unrelated conditions [22, 27, 31].

**Fig. 1:**
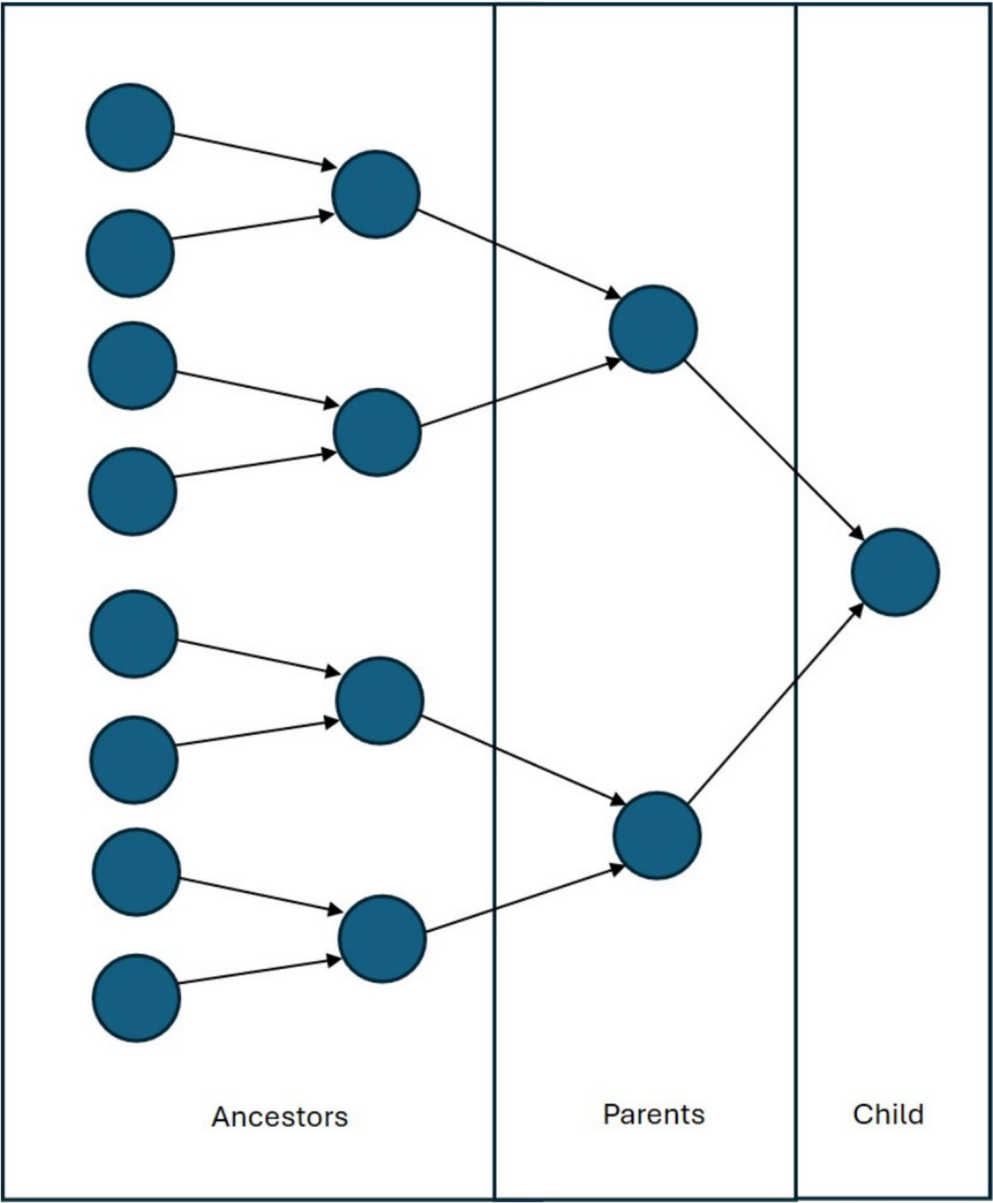
A causal DAG reflecting a family tree and the transmission of genetic information through the ancestral lineage. Despite all ancestors of the parents having a (indirect) causal effect on the child’s genetics, these effects are mediated through the child’s parents, and all relevant ancestral genetic data required to predict the genetics of the child are therefore captured in the parents. Accordingly, if the genetic composition of the parents is known, including additional genetic information from the ancestors in a prediction model is redundant and likely to add unnecessary noise. *DAG* directed acyclic graph

To provide a relatable analogy of this that aligns with the ancestral terminology used in causal graphs, the passing of genetic information to a child through a family tree is useful (Fig. 1). Here, the parents are the child’s direct (most proximal and unmediated) causes (Table 1) that ultimately determine the child's genetic composition. In contrast, ancestors such as grandparents and great-grandparents are indirect causes (Table 1) because their effects are mediated (Table 1) through the parents, that is, the parents are the mechanism transmitting ancestral effects to the child. For the purposes of prediction, the critical implication is that, and according to the causal Markov condition, all relevant genetic material needed to predict (and produce) the child is captured in the parents, and including additional genetic information obtained from their ancestors is entirely redundant. This is not to say that, for the purposes of athletic injury prevention, the more distal ancestor variables are irrelevant. One may certainly intervene on distal causal variables, such as nutrition, sleep, coordination etc. to have a causal effect on an outcome of interest such as injury or athletic performance [32]. However, for the purposes of prediction (which is a separate problem to causality and intervention [32]), including these more distal causal variables within a prediction model is redundant if the more proximal variables already containing the relevant statistical information are included in the model. This has the advantage of both simplifying the model and reducing noise [22, 27, 31].

For athletic injuries, the most fundamental causal variables are the mechanical loads that are applied to a tissue (and the stresses and strains they induce), the tissue’s capacity to withstand these loads (mechanical strength), and the accumulation of tissue damage over time (whether directly mechanically induced or also as a function of the repair and remodelling process) [8, 32–35]. According to the causal Markov condition, all distal causal variables (e.g., nutrition, sleep, coordination, etc.) must exert their influence on injury risk through their effects on either the strength of an at-risk tissue, the loading experienced by that tissue, or both. It follows that these variables jointly contain all relevant statistical information required to predict injury. Thus, their accurate quantification, together with a mathematically precise definition of athletic injury (as presented in Sect. 3), provides the foundation for models capable of predicting when an injury will occur [8].

**Fig. 2:**
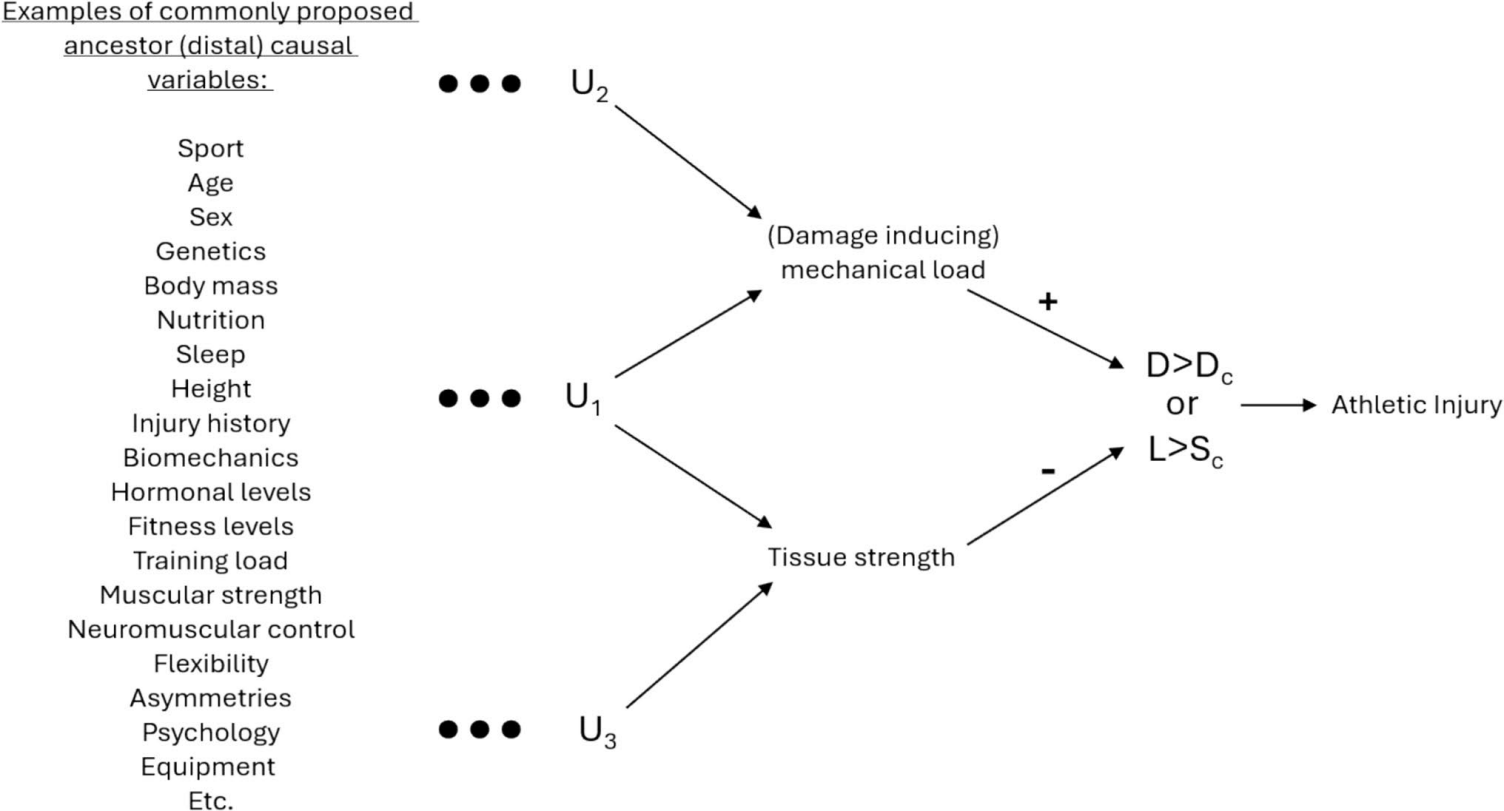
Reflecting the ancestral lineage scenario in Fig. 1, a signed causal DAG [95, 96] depicting mechanical loading (*L*) exceeding a critical tissue strength threshold (*S*_c_) (i.e., *L* > *S*_c_) or as an alternative conceptualisation, damage (*D*) exceeding a critical damage threshold (*D*_c_) (i.e., *D* > *D*_c_) as the ultimate mechanism of acute athletic injury occurrence. The ( +) symbols indicate that increased values of a variable result in increased values of the variable being affected, e.g., larger mechanical loads result in an increased risk of *L* > *S*_c_ (or *D* > *D*_c_). The (–) symbols indicate that increased values of a cause result in decreased values of the variable being affected, e.g., higher values of tissue strength result in a decreased risk of *L* > *S*_c_ or *D* > *D*_c_. A generic wider causal structure (ancestor variables) is included for illustrative purposes with the ‘U’ variables representing unspecified ancestor variables and the ellipses alluding to the existence of a wider causal network, such as genetics, age, nutrition, sleep, etc. Crucially, all distal variables must exert their influence on injury risk through their effects on either mechanical loading (*L*), tissue strength (*S*_c_), or both. Accordingly, all information required to predict whether an acute athletic injury occurs is, in principle, captured within these key variables. Specifically, when *L* > *S*_c_ or *D* > *D*_c_, an athletic injury has occurred. However, for injuries of this nature, predictions using these variables may provide no meaningful time window for intervention. Adapted from Kalkhoven 2024 with permission [32]. DAG directed acyclic graph

For acute athletic injury, a causal DAG is presented in Fig. 2. This DAG illustrates that a single mechanical load applied to a tissue, together with the tissue’s mechanical strength, are the proximal variables that causally determine whether an acute injury occurs. Specifically, if the applied load exceeds the tissue’s critical strength threshold, producing damage beyond a critical limit, an acute injury results. An example is a bone fracture in soccer following a poorly timed tackle, where the ultimate strength of the bone is exceeded by a single impact load. By accurately quantifying these proximal variables, the occurrence of such injuries can, in principle, be predicted. In practice, however, such prediction will likely provide no meaningful time window for intervention. Nevertheless, this framework can still be used to inform injury intervention strategies, for example, simulating how different helmet types in Formula One racing reduce brain loading under crash conditions, or how shinpads reduce the risk of bone fracture in soccer. It should also be emphasised that considerable evidence indicates many tissue failures in sport, often classified as acute athletic injuries such as Achilles tendon ruptures, anterior cruciate ligament tears, and hamstring strains, are not always isolated traumatic events, but instead frequently reflect underlying pathological processes [91–94].

**Fig. 3:**
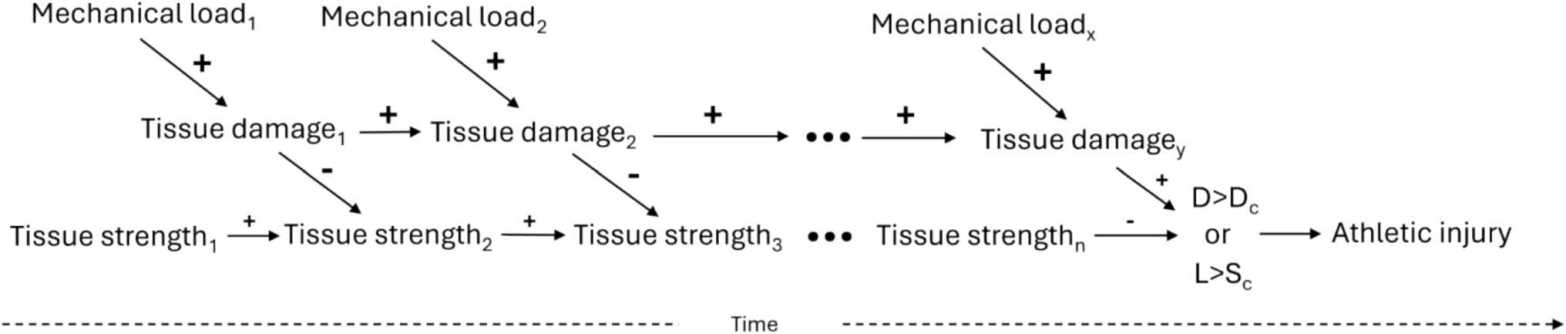
A signed causal DAG [95, 96] depicting repetitive mechanical loading as a cause of tissue damage and the deterioration of tissue strength (mechanical fatigue), which eventually results in gradual onset injury occurrence. The subscript values represent the sequence of events, e.g. the tissue of interest has its original strength (tissue strength_1_) prior to a mechanical load (_1_) being applied, which results in tissue damage (tissue damage_1_). This causes a reduction in tissue strength (_2_) owing to mechanical fatigue. A second subsequent mechanical load (_2_) is applied, which causes further damage (_2_) and a further reduction in tissue strength (_3_). This process continues until a critical threshold is exceeded and injury occurs. The ( +) symbols indicate that increased values of a variable result in increased values of the variable being affected, e.g., larger mechanical loads result in greater tissue damage being experienced. The (-) symbols indicate that increased values of a cause result in decreased values of the variable being affected, e.g., higher amounts of tissue damage result in larger decreases in tissue strength. The ellipses in the figure represent a time jump to the point where injury occurs. Note: Physiological processes, i.e., remodelling and repair, have been intentionally omitted from this DAG for simplicity. Adapted from Kalkhoven 2024 with permission [32]. *DAG* directed acyclic graph

In Fig. 3, a causal DAG for gradual onset injuries is presented, whereby the accumulation of damage due to repetitive loading damages a tissue over time, reducing its mechanical strength in the process. When the damage sustained exceeds a critical damage threshold, or as an alternative conceptualisation, a final mechanical load (in a sequence of loads) exceeds a declining critical tissue strength threshold, a gradual onset injury occurs. Accordingly, for gradual onset injury, an athletic injury can be predicted through quantifications of either the damage sustained and a clearly defined critical damage threshold or of mechanical loading and the (deteriorating) strength of the tissue.

## Modelling of Load, Strength, and Damage Interactions for Athletic Injury Prediction

### Predicting Athletic Injuries with a Probability of Tissue Overload Analysis

While determinism offers the allure of perfect predictability and precision, and is valuable for precisely defining a concept for mathematical modelling, in real-world settings deterministic modelling is often a theoretical ideal rather than an attainable practical reality. This is typically a result of the complexities and uncertainties that exist in real-world settings. For example, perfectly measuring any variable is beyond current technological capabilities, and accordingly, there will always be some degree of measurement or estimation uncertainty that needs to be accounted for. To account for these uncertainties, probabilistic models are commonly used [35, 97–101]. For athletic injuries, predicting the occurrence of an athletic injury centres around *D* > *D*_c_ or *L* > *S*_c_.

Accordingly, by quantifying or estimating these variables, the probability that an athletic injury has occurred can be calculated.

**Fig. 4:**
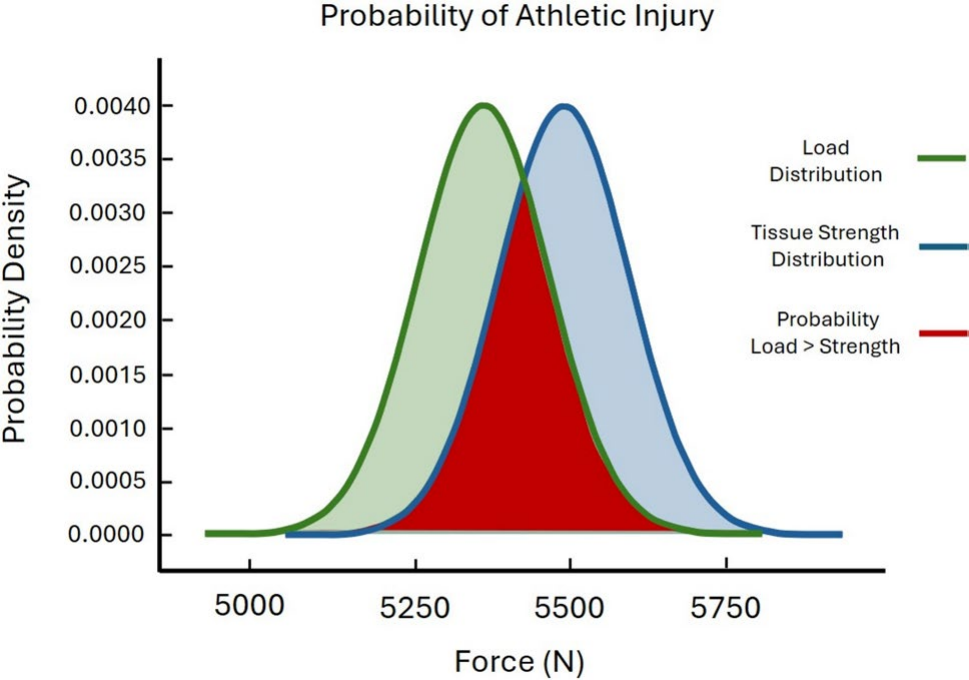
Probability of injury (simplified to tissue failure) calculated using normal distributions of load and strength estimates, accounting for uncertainty in variable estimation. By scrutinising the overlap of probability density functions for these variables, the risk of injury or tissue failure can be appropriately quantified

To provide an example of this (Fig. 4), and for simplicity’s sake, the critical tissue strength threshold will be assigned as the failure force (measured in Newtons) of the tissue, which represents the force at which complete tissue rupture or fracture occurs, corresponding to a damage variable of 1. As there will inevitably be some uncertainty when estimating this tissue strength threshold, distributions can be used to depict this (Fig. 4). Similarly, a distribution can also be used to represent the tissue loading estimate (Fig. 4). If the uncertainty is high, the distributions will be wider, reflecting increased uncertainty in the actual values of these variables. If the uncertainty is low, the distributions will be narrower, reflecting decreased uncertainty in estimation. From here, the overlap of probability density functions (depicted in red in Fig. 4) for these variables can be scrutinised, and the probability that the applied load exceeds the mechanical strength of the tissue (i.e., that an injury occurs) can be appropriately quantified.

Note, the presented example, while accurate, is in many contexts a simplification of the problem, as tissue strength is relative to the type of loading applied (e.g., tensile, compressive, shear etc.). Accordingly, this model assumes a particular type of load and corresponding strength response. Many biological tissues are inherently anisotropic (Table 1) due to their complex architecture [102–104], while muscle is also a sophisticated active tissue whose mechanical behaviour in response to loading is dependent upon muscle activation patterns and the neuromechanical regulation of tensions and stiffness [105–108]. It follows that, the stress and strain experienced by specific regions of a musculotendinous unit presents a particularly complex phenomenon to model [105–109].

### Simulation of Damage Accumulation and Tissue Strength Deterioration to Failure: A Case Example Using Achilles Tendon Deterioration to Rupture and Repeated Measures

**Fig. 5:**
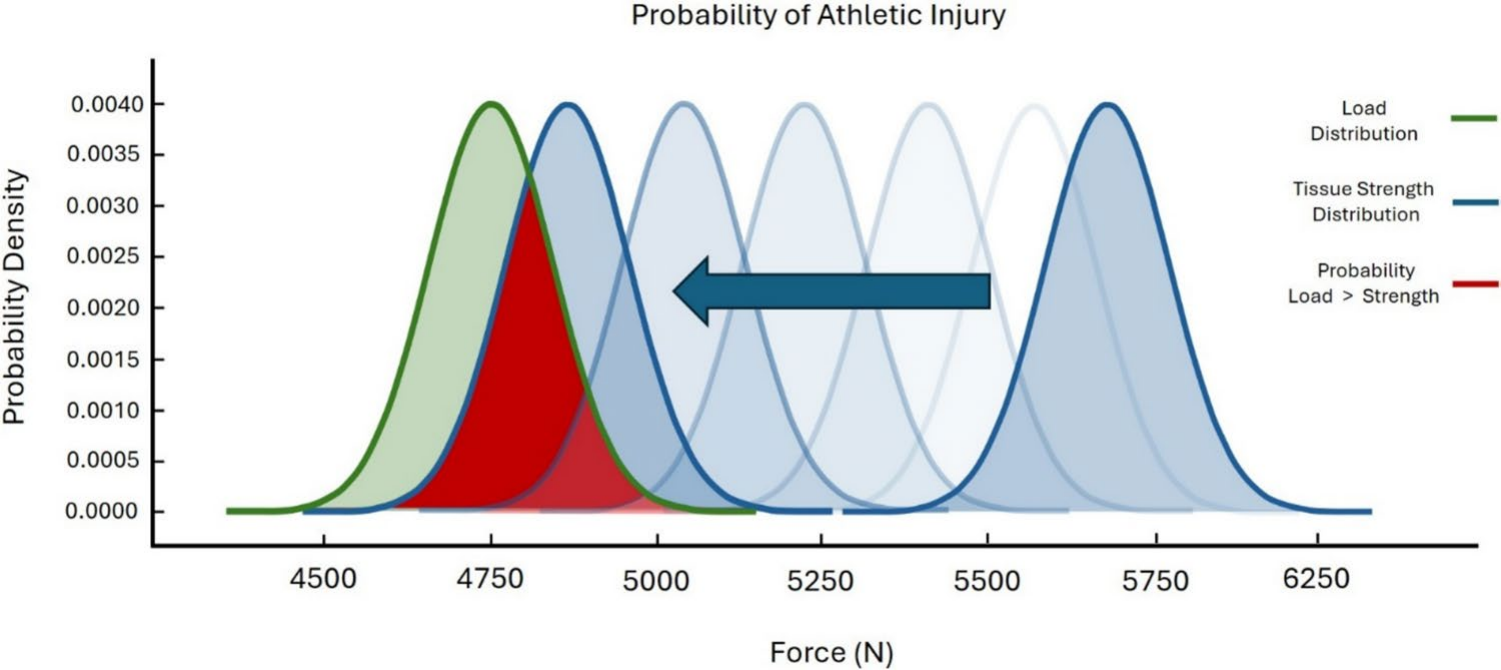
Simulation of Achilles tendon rupture risk for a deteriorating tendon using tendon load and repeated strength estimates. Achilles tendon load is represented in green and tissue strength is represented in blue. The normal distributions reflect the uncertainty in variable estimations. With each new measure of tendon strength (reflecting new measures over various time intervals), a reduction in tendon strength is observed, as highlighted by the leftward shifting bell curves in various shades of blue. The probability of tendon failure is calculated by scrutinising the overlap of probability density functions for Achilles tendon load and strength estimates

To provide an example of a probability of athletic injury analysis for a deteriorating tissue, a probability of rupture analysis (corresponding to a damage threshold of (1) for a gradually weakening Achilles tendon is presented (Fig. 5). For simplicity, the estimated load remains stable and can be conceptualised as the maximum (tensile) Achilles tendon load experienced by an athlete when competing. Conversely, tendon strength (*S*_f_) deteriorates over time, with this reduction in strength being captured by repeated tissue strength measures taken at different time points (as indicated by the leftward shifting distributions in blue). As the Achilles tendon is losing strength over time while the load remains constant, load and tissue strength values converge after each new measure. Note that while the Achilles tendon was chosen for this example [92, 93], ultimately, similar approaches can be adopted for any tissue across the human body (e.g., ligaments such as the anterior cruciate ligament [91], bone, muscle etc. [36]), although the dynamic nature of musculature and muscle–tendon functioning may add some additional complexities [105–109].

For this example,* S*_c_ is set to the tendon failure strength (*S*_f_) for ease of conceptualisation, with an initial estimate of 5659.1 ± 100 N [110]. The applied load is estimated at 4750 ± 100 N. For simplicity, the load estimate is kept constant. Repeated measures of tissue strength are taken over time, with each measure reflecting a deterioration in tendon strength (represented by the leftward shifting normal distributions of strength towards the load estimate). This shift in tendon strength follows the following trajectory between measures: tendon strength_1_ = 5659.1 ± 100 N, tendon strength_2_ = 5574.3 ± 100 N, tendon strength_3_ = 5391.2 ± 100 N, tendon strength_4_ = 5223.8 ± 100 N, tendon strength_5_ = 5039.2 ± 100 N, tendon strength_6_ = 4873.5 ± 100 N. As the strength deteriorates and moves closer toward the load, eventually the overlap of the probability density functions of tissue strength and load becomes increasingly visible (depicted in red in Fig. 5). This starts at tissue strength_4_, with an increasing overlap at tissue strength_5_ and tissue strength_6_. It is this overlap that allows quantifications of the probability of an Achilles tendon rupture occurring (i.e., *L* > *S*_f_).

To demonstrate how the probability of an Achilles tendon rupture can be calculated from these data, a calculation using the overlap between load and tissue strength_6_ is presented. First, the distribution of the difference between the load and the strength estimates needs to be determined. To achieve this, the following notations are used: load (*L*), strength (*S*_f_), normal distribution (*N*), mean estimate (*μ*), and probability (*P*).

Given:

- *L* ~ *N*(4750, 100^2^)
- *S*_f_ ~ *N*(4873.5, 100^2^)

The difference (Diff) = *L* – *S*_f_ will also be normally distributed with the mean and variance given by:

- Mean (*μ*) of Diff, *μ*_D_ = *μ*_L_ – *μ*_Sf_ = 4750 − 4873.5 =  − 123.5
- Variance of Diff,* σ*^2^_D_ = σ^2^_L_ + σ^2^_Sf_ = 100^2^ + 100^2^ = 20,000
- Standard deviation of Diff, * σ*_D_ = $\sqrt{20000}$  = 141.42

Now, Diff ~ N(− 123.5, 141.42^2^).

Find *P*(*L* > *S*_f_), which is equivalent to *P*(Diff > 0).

To find this probability, the *Z*-score for Diff = 0 is calculated:

$$Z = \frac{{0 - \left( { - 123.5} \right)}}{141.42} = \frac{123.5}{{141.42}} \approx 0.{8732}$$

Using the Z-score, the probability that a standard normal variable is less than 0.8732 is approximately 0.8088.

Therefore, the probability that Diff > 0 is:

$$P \, \left( {D \, > \, 0} \right) \, = \, P \, \left( {Z \, > \, 0.8732} \right) \, = \, 1 - 0.8088 \, = \, 0.1912$$

**Fig. 6:**
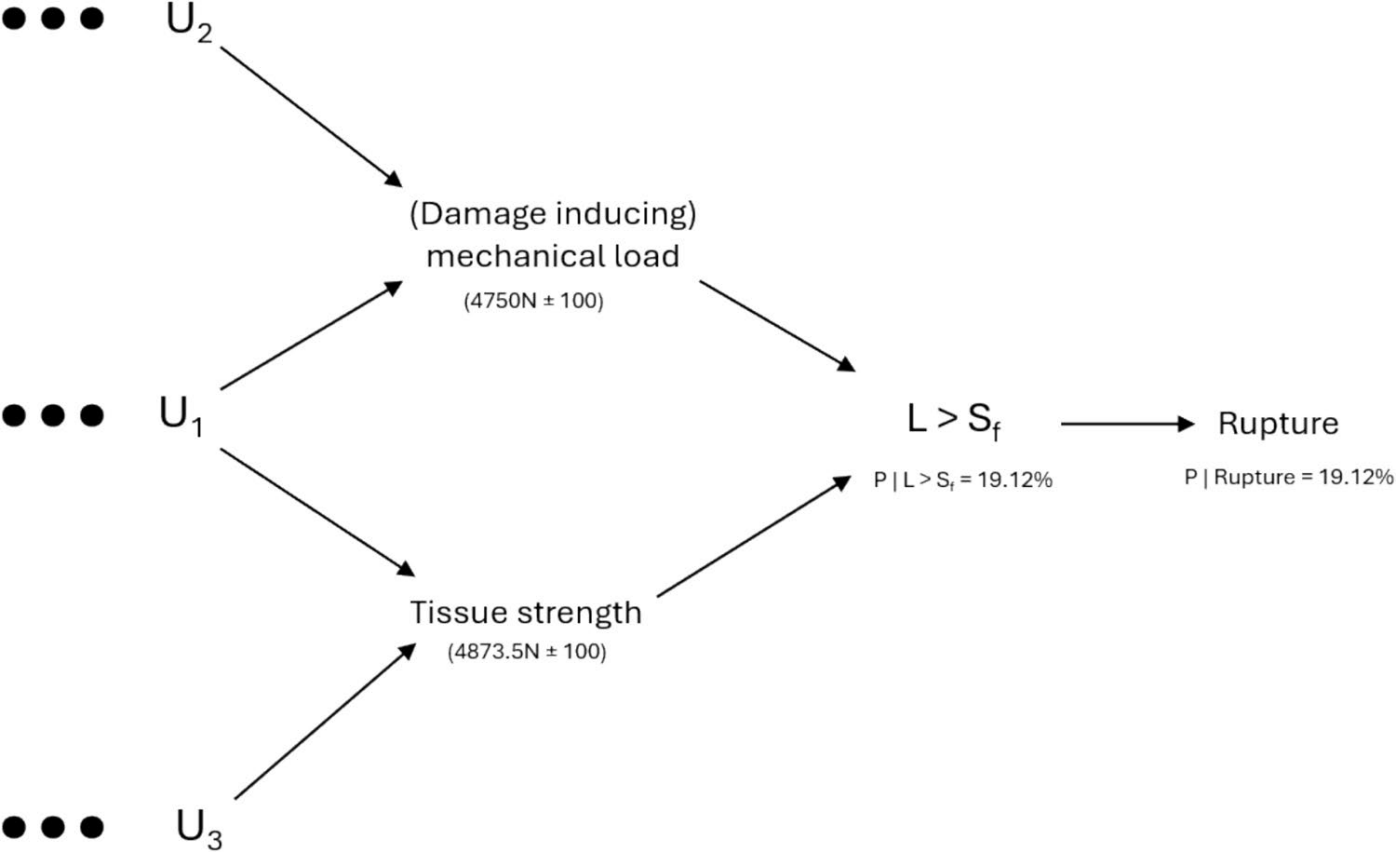
A causal DAG presenting a probability (*P*) of Achilles tendon rupture based on estimations of mechanical load (*L*) and tendon failure strength (*S*_f_). A generic wider causal structure (ancestor variables) is included for illustrative purposes with the ‘*U*’ variables representing unspecified ancestor variables and the ellipses alluding to the existence of a wider causal network beyond that presented in the DAG (e.g., genetics, age, nutrition, sleep etc.)

Accordingly, for this example, the probability that the estimated Achilles tendon load is greater than tendon strength_6_, and an Achilles tendon rupture occurs, is approximately 0.1912, or 19.12%. This example can also be presented in causal DAG form, as presented in Fig. 6.

## Theory to Practice: Intervention, Estimation, and a Call for Innovation

To offer practical utility for the prevention of athletic injuries, injury predictions must provide sufficient windows of opportunity for intervention. However, for injuries involving sudden, unanticipated loads that exceed the possible physiological limits of tissue strength, the predictive methods explored in this article may be of limited value, as they do not offer actionable lead times for intervention. In such acute injury scenarios, a shift in focus toward more distal variables influencing injury occurrence, such as biomechanical movement patterns, situational patterns, and wider contextual factors, may offer greater potential for intervention, although current evidence supporting their predictive utility remains limited [6]. Nevertheless, even when real-time prevention is not feasible, the models proposed within this article may still contribute value through simulation, informing system-level decisions and long-term injury prevention strategies.

By contrast, in scenarios where future loads experienced by an athlete can be appropriately estimated, tissue damage accumulates over time, or tissue load and strength values converge over time, obtaining accurate estimates of the most immediate precedents determining athletic injury outcomes may provide powerful athletic injury risk assessments that offer specific data-driven windows for intervention. While this approach is theoretically strong, mimicking the processes through which mechanical engineers predict damage accumulation, defect formation, lesion formation in biological tissues, and mechanical failures [35, 83, 88, 111], the effective application of this approach in real-world settings is reliant upon the accurate estimation of tissue loading, strength and damage accumulation in vivo—a task currently complicated by limitations in measurement technologies and analysis methods. Accordingly, in the following section, potential avenues for estimating these critical variables with the necessary accuracy needed to actualise the proposed approach of athletic injury prediction in real-world settings will be explored.

### Estimating and Modelling Damage Accumulation and Injury Risk Through the Mechanical Loading Pathway in Applied Sports Settings: Progress and Limitations

In a 2018 article, Edwards [35] provided an important perspective on damage accumulation in biological tissues and the modelling of overuse athletic injuries. Some key points from this article included the importance of understanding the effects of load magnitude on mechanically induced tissue damage, whereby small increases in load magnitude can drastically reduce the number of loading cycles required for tissue failure, even when cumulative loads are similar. The implications of this are that the loading pattern (i.e., specific combinations of load magnitude and cycles) plays a crucial role in damage accumulation, and cumulative load metrics, as commonly applied across the sporting world, provide an inadequate basis for modelling damage accumulation and assessing athletic injury risks. One such method commonly used in materials sciences to account for the complex interaction between load magnitude and loading cycles is the Palmgren–Miner rule (or Miner’s rule; Table 1) [48, 49], a relatively simple linear damage accumulation model used to predict mechanical failures. Supplementary to this is the common use of probabilistic approaches to account for the inherent variability in material responses to loading. The Weibull distribution (Table 1), for example, is often used to model the variability in material fatigue behaviour, offering a probabilistic framework to estimate the likelihood of tissue failure under different loading conditions [54, 112, 113]. While further exploration of methods used for mechanical fatigue damage modelling is certainly warranted, this has already been outlined in existing literature [35]. Accordingly, for a deeper understanding of these methods as they apply to fatigue damage modelling in biological tissues, the reader is directed to other published literature on the topic [35–37], and the practical utility of these methods will be explored herein.

For the effective application of common fatigue damage modelling methods for athletic injury prediction, accurate estimations of musculoskeletal loading appear crucial. From a causality perspective, and as previously noted, the application of mechanical loads to a tissue is fundamental to athletic injury occurrence, overloading the tissue either through the application of a single load that exceeds the strength of a tissue or through repetitive loading cycles that damage the tissue over time (Figs. 2 and 3). It follows that, by accurately estimating the mechanical loading placed on specific tissues, the overload and accumulation of damage that ensues can be modelled using various methods, such as those already discussed. One study seeking to bridge the gap between theory and practice utilising this approach is that of Matijevich et al. [114]. In their study, these authors leveraged a combination of wearable sensor signals, machine learning, and biomechanics to develop multi-sensor algorithms to estimate tibial bone forces and cumulative damage (adopting the previously discussed Miner’s rule) when running, which is a common site for gradual onset injury in runners (i.e., tibial stress fractures). In this study, the performance of vertical average loading rate (VALR), a common impact measure derived from inertial measurement units, was compared with a physics-based algorithm and a machine learning algorithm. Notably, VALR resulted in a mean absolute percent error of 9.9% when estimating tibial forces, while the physics-based and machine learning algorithms reduced errors to 5.2% and 2.6%, respectively. Although these errors may appear low, these authors emphasised that relatively modest errors in tibial force estimates translated into large errors when estimating bone damage. Specifically, a 9.9% error in tibial force using VALR translated into a 104% error when estimating bone damage, while the physics-based and machine learning algorithms reduced damage errors to 41% and 18%, respectively, highlighting the importance of obtaining highly accurate estimates of mechanical loading when utilising this approach. Since the release of this article [114], further studies adopting similar approaches have been presented in the literature [115, 116].

While the modelling of athletic injury through the mechanical loading pathway has yielded some promising results [114], there remain a number of practical limitations that might restrict the effective application of this approach for athletic injury predictions in the applied sporting world. To accurately model the accumulation of damage to failure, an accurate estimate of the mechanical strength of a tissue is typically required. Simple cumulative damage estimation methods such as Miner’s rule also adopt a number of assumptions and do not take into account the order in which different stress levels are applied (although various non-linear and sequence-dependent damage models that do take into account the order of stress application have been developed [117, 118]), nor were they developed to account for the tissue remodelling and repair processes. In addition, current approaches to estimating musculoskeletal loading rely upon the fusion of multiple wearable sensors, which need to be comfortable and non-intrusive to ensure athletes can utilise them unimpeded. To be practically viable, these technologies must be seamlessly integrated onto the athlete without negatively impacting them or the estimates of mechanical load and tissue damage that are critical for injury risk assessments. This may prove particularly challenging if multiple injury sites are of concern, as the setup utilised in the study by Matijevich et al. [114] (which included sensors on the foot, shank, and insoles) was adopted for one specific location, the tibia.

Some potential avenues that may assist in this regard are the further development of minimalist wearable setups utilising smaller and/or fewer devices, or the integration of markerless motion capture, which has shown some potential for estimating tissue forces during various motor tasks [119]. However, this technology is in its infancy [120]. Finally, an additional challenge to overcome is the accurate estimation of in-vivo muscle forces, which is a particularly complex phenomenon to model. Indeed, the mechanical behaviour of this tissue is highly dynamic and dependent upon the degree of activation, and the alterations in active muscle tensions and stiffness that ensue [105–108]. It follows that current computational models (primarily Hill-type models) still do not accurately predict muscle forces under dynamic conditions [121–123], although innovative approaches to solving this problem are being developed [107, 108]. While the modelling of tissue damage through the mechanical loading pathway demonstrates some promise, when considering that for some tissues (such as the tibia when running), the majority of mechanical loading is derived from muscle contraction [114], and that relatively small errors in load estimation propagate into large errors in cumulative damage estimations [114], exploration of alternative approaches to athletic injury prediction is warranted.

### Tissue Strength and Damage Assessments: Scanning Technologies, Artificial Intelligence, and Mechanical Testing

Medical scanning technologies have revolutionised the field of healthcare by providing non-invasive methods to visualise the internal structures of the human body. Among the most prominent scanning technologies are magnetic resonance imaging (MRI), X-ray, computed tomography (CT), ultrasound, and positron emission tomography (PET). Notably, medical imaging is one such area that artificial intelligence is poised to revolutionise. Traditionally, the interpretation of medical images has been performed by human experts such as radiographers and sonographers. However, with the emergence of artificial intelligence, this technology now enables sophisticated pixel-level analysis, which can detect subtle patterns and anomalies that have historically been imperceptible to the human eye. Indeed, artificial intelligence models have demonstrated remarkable performance outcomes on certain medical imaging-based tasks, significantly enhancing diagnostic accuracy and efficiency in some contexts [124, 125], while also providing exceptionally high prediction capabilities on tasks that clinical experts have previously struggled with [126, 127], even from corrupted, cropped, and noised medical images [127].

The capacity for artificial intelligence to analyse medical scans with unprecedented capabilities holds significant potential for athletic injury prediction. By detecting subtle changes in tissue characteristics, the merging of scanning technologies with artificial intelligence may potentially identify tissues at heightened risk of injury or rupture based on the identification of critical tissue features preceding athletic injury occurrence, and by obtaining effective estimates of tissue strength and damage. Supporting this promise, recent work by Cushman et al. [128] demonstrated that even conventional ultrasound-detected abnormalities in the patellar tendon, Achilles tendon, and plantar fascia were associated with substantially elevated time-loss injury risk, underscoring the potential prognostic value of subtle structural changes. Similarly, other researchers have begun exploring alternative imaging modalities, such as shear wave elastography, to predict ligament failure risk [129]. However, while the identification of certain tissue features of interest may prove to be of value for athletic injury predictions, keeping with the approach of injury risk assessment explored within this article (Fig. 5), there is a specific interest in obtaining quantified mechanical strength estimates from these technologies. This is a challenging endeavour as the mechanical strength of a tissue is determined by a wide array of variables including tissue shape, size, architecture, and material properties [130]. At present, the most direct and reliable method for determining tissue strength involves destructive mechanical testing (Table 1), typically by pulling the tissue to failure under controlled conditions [110, 111]. Of course, this is not a viable option for athletes, and accordingly, exploration of alternative methods that may potentially assist with obtaining useful estimates of this variable in vivo is needed.

One potential solution is the utilisation of mechanical strength values obtained through mechanical testing as training data for artificial intelligence models. To elaborate, artificial intelligence models appropriately trained on medical imaging and mechanical strength data obtained from ex vivo mechanical testing may be able to predict the mechanical strength of tissues in vivo [131–137]. This would also allow for in vivo estimates of tissue damage, which can be calculated from repeated strength estimates (Eq. 1). However, several logistical challenges exist. The scanning technology adopted may require adequate resolution, contrast, and signal quality to sufficiently capture the tissue’s internal microarchitecture [133, 138]. Additionally, image-based models typically require large datasets (often thousands of images) [133, 139], and obtaining matched strength data demands many tissue samples, which is particularly problematic if human tissue samples are to be used. Nevertheless, if a model is trained upon a large and varied-enough set of tissue samples (e.g., multiple animal tissues), a single model may potentially generalise to multiple tissues across the human body [140].

Ultimately, the practical utility of such an approach may be dependent upon a number of factors, such as the specific tissue type, its location, and compatibility between the adopted scanning technology and the tissue type being assessed. In addition, collecting this type of data on field is currently unfeasible, and accordingly, injury risk analyses utilising this approach would be reliant upon opportunity windows for tissue assessment, such as those between sporting exposures. Despite this, integrating artificial intelligence with advanced imaging remains a promising strategy for assessing tissue health, strength, and damage. By providing more direct estimates of tissue properties, this approach may bypass the need for modelling through the mechanical loading and damage accumulation (and removal, i.e., tissue remodelling and repair) pathway, avoiding the associated error propagation in cumulative damage estimations when using this method and potentially offering a more powerful basis for athletic injury prediction.

### Assessing the Mechanical Behaviour and Properties of Tissues to Model Damage Accumulation and Injury Risk

While an artificial intelligence model effectively trained to estimate tissue strength and damage could offer powerful injury risk assessments in certain contexts, alternative approaches focusing on other mechanical properties may provide a more feasible or complementary means of predicting injury risk. Mechanical damage not only reduces tissue strength but also induces measurable changes in properties such as tissue strain, stiffness, and hysteresis (Table 1) [51, 111, 141]. Accordingly, such variables may act as appropriate proxy measures of tissue strength and damage in certain contexts [51, 111, 141]. Notably, Fung et al. [51] found that tendon strain increased progressively and irreversibly with microdamage accumulation in cyclically loaded rat tendons, preceding significant changes in stiffness and hysteresis, which were only observed at high levels of mechanical fatigue. This highlights strain as a particularly sensitive and early indicator of tissue degradation. In bone, Burr et al. [141] found that reductions in elastic modulus precedes the accumulation of microscopically visible damage, with significant microcrack formation emerging after ~ 15% stiffness loss. Beyond this threshold, damage area increased linearly with further modulus degradation. Perhaps most notably, Firminger and Edwards [111] found that in load-controlled mechanical testing of the human patellar tendon, peak stress and initial peak strain correlated with fatigue life (i.e., the number of load cycles to failure for a given stress level) (*r*^2^ = 0.65 and *r*^2^ = 0.57, respectively; *p* < 0.001). However, the rate of peak cyclic strain increase (creep rate) and Young’s modulus degradation rate (commonly termed damage rate) displayed remarkably strong correlations with fatigue life (*r*^2^ = 96% and *r*^2^ = 86%, respectively; *p* < 0.001). Collectively, these findings suggest that non-invasive monitoring of tendon strain behaviour and stiffness degradation may serve as sensitive and quantitative indicators of tissue integrity and mechanical capacity, potentially offering highly predictive markers for gradual-onset injury and tissue failure occurrences.

By assessing multiple tissue parameters such as progressive tissue strain profiles, stiffness alterations, and microstructural changes, there is potential to accurately estimate tissue strength, damage accumulation, injury risk, and risk of failure in vivo. While this approach could provide critical insights into the progression of tissue damage over time, enabling timely interventions for injury prevention, several challenges remain. As this approach is largely reliant upon scanning technologies, logistical constraints exist, particularly regarding on-field tissue assessments. Additionally, lab-based data collection can be time consuming, often requiring rigorous setups and some degree of tissue loading and deformation during scanning [142, 143]. Despite these challenges, promising innovations in various technologies (such as wearable ultrasound patches [144, 145] and shear wave tensiometers [146]) are emerging in the literature. Advancements such as these could help overcome existing limitations, improving the feasibility of real-time, non-invasive tissue monitoring for injury risk assessment and prevention.

## Conclusion

In the realm of competitive sports, the prediction of athletic injuries remains a critical goal that demands accuracy and innovation. This article aimed to provide an underlying framework, developed from first principles, for achieving effective athletic injury predictions for certain types of injury, and to explore potential methods for real-world actualisation. By establishing (and mathematising) a fundamental theoretical definition of athletic injury, understanding and leveraging critical causal variables, and appropriately employing probabilistic models, the quest for highly accurate and actionable athletic injury predictions may be achievable. However, the real-world application of the approaches explored in this article require advancements in measurement technologies and data integration. Specifically, innovative developments in and utilisation of wearable technology, medical imaging, and artificial intelligence appear the most likely avenues to assist with achieving high-accuracy athletic injury predictions for application in the applied sporting world. However, while these approaches provide exciting research pathways, ultimately, those interested in solving existing athletic injury prediction problems should not feel restricted to these suggestions. Rather, researchers are encouraged to explore whichever innovative solutions they see fit but are guided toward attempting to obtain accurate estimates of tissue loading, damage, and strength. This is advocated with the understanding that, for certain types of injury, the accurate measurement or estimation of these variables appears critical in the quest for accurate and actionable athletic injury predictions, reflecting a fundamental problem in measurement and estimation rather than data analytics and complex systems, despite the latter being a common emphasis in sports science and medicine literature.
